# Impact of Music in Males and Females for Relief from Neurodegenerative Disorder Stress

**DOI:** 10.1155/2022/3080437

**Published:** 2022-04-12

**Authors:** Nilima Salankar, Anjali Mishra, Deepika Koundal, Vinh Truong Hoang, Kiet Tran-Trung, Atef Zaguia, Assaye Belay

**Affiliations:** ^1^School of Computer Science University of Petroleum and Energy Studies, Bidholi, Dehradun, India; ^2^Faculty of Computer Science, Ho Chi Minh City Open University, Address: 97 Vo Van Tan Ward Vo Thi Sau District 3, Ho Chi Minh City, Vietnam Code Postal: 70000; ^3^Department of Computer Science, College of Computers and Information Technology, Taif University, P.O. BOX 11099, Taif 21944, Saudi Arabia; ^4^Department of Statistics, Mizan-Tepi University, Tepi, Ethiopia

## Abstract

Neurological imbalance sometimes resulted in stress, which is experienced by the number of people at some moment in their life. A considerable measurement scheme can quantify the stress level in an individual, in which music has always been considered as the best therapy for stress relief in healthy human being as well in severe medical conditions. In this work, the impact of four types of music interventions with the lyrics of Hindi music and varying spectral centroid has been studied for an analysis of stress relief in males and females. The self-reported data for stress using state-trait anxiety (STA) and electroencephalography (EEG) signals for 14 channels in response to music interventions have been considered. Features such as Hjorth (activity, mobility, and complexity), variance, standard deviation, skew, kurtosis, and mean have been extracted from five bands (delta, theta, alpha, beta, and gamma) of each channel of the recorded EEG signals from 9 males and 9 females of the age category between 18 and 25 years. The support vector machine classifier has been used to classify three subsets: (i) male and female, (ii) baseline and female, and (iii) baseline and male. The noteworthy accuracy of 100% was found at the delta band for the first subset, beta and gamma bands for the second subset, and beta, gamma, and delta bands for the third subset. STA score has shown more deviation in the male category than in female, which gives a clear insight into the impact of music intervention with varying spectral centroid that has a higher impact to relieve stress in the male category than the female category.

## 1. Introduction

Any changes in the external or the internal environment that demands the human body to react and adjust often lead to stress that has usually been found in the college students. The external environment such as studies, job, society, and relationships are major impacting and internal environment like own thoughts and arised emotions with respective to thoughts. According to Alison Abbott, the stress can be threatening to both physical as well as mental health [1]. Stress causes many mental health problems such as hypertension [2], stroke [3], sleep disturbances [4], and depression/suicide [5]. It has also been found stress is one of the elements causing physical disorders such as cardiac attack [6], irritable bowel syndrome (IBS), back pain, and gastro-oesophageal reflux disease (GERD) [7]. Therefore, several responses have been used to measure the stress level. Clinically, the stress level of an individual is evaluated by the self-reported questionnaires with the rating scale such as relative stress scale, perceived stress scale (PSS) [8], and state-trait anxiety inventory (STAI) [9]. Apart from this, the physical features of an individual can also be used as one of the methods to compute and measure the stress such as facial expressions [10] and blink rate [11]. The stress causes dynamic alterations in the autonomous nervous system [12]. Also, it impacts the heart rate [13], skin conductance [14], and respiration [15]. According to neurologists, the stress majorly affects the human brain as it has a tendency to demonstrate the stressful situations [16, 17]. The electroencephalography (EEG) [18] and functional magnetic resonance imaging (FMRI) [19] are most commonly used tools to examine the brain activity. The EEG is the most preferred method because of inexpensive equipment with less disruption. In reference [20], the responses such as heart rate variability (HRV) and heart rate are very much associated with EEG in response to the stress.

Looking onto the effects caused by the stress on an individual's life, it is required to uncover the approaches, which could help to get relief from the stress and recuperate to the mental stability. Among the ample available approaches, music has been found as one of the therapies used for the mental disability treatment because of its power to change emotions and psychological behaviour of the human being [21, 22]. Listening to music is very common among the people for the mental relaxation in general [23, 24]. Moreover, it also helps to get into relaxing mode during anxiety or stressful situations irrespective of age and sex [25]. This therapy works very well on human beings [26, 27]. It plays a vital role in an individual's daily life for stress releasing as it directly impacts the brain activity [28]. Various methods have been used for visualizing the characteristics of the signals [29, 30]. Hippocampus and amygdala are the two brain structures, which are activated during the stressful situation, are known to be concerned with the regulation of the hypothalamic-pituitary-adrenal (HPA) axis, and are responsible for the tempting emotions with the music [31]. Another part of the human body, which is sensitive to the stress, is ANS, which provokes the physiological changes in response to music. Hence, this is the reason to study the impact of listening to music on the stress and anxiety levels extensively. According to various studies, music has a tendency to improve the emotional health of an individual by lowering down the stress level. The electrical signals generated in the human brain are captured by the EEG through the electrodes placed on the scalp. These EEG signals vary in the frequency and amplitude. Generally, the EEG signal frequency is classified into five frequency bands as delta (*δ*: up to 4 Hz), theta (*θ*: 4–8 Hz), alpha (*α*: 8–15 Hz), beta (*β*: 15–32 Hz), and gamma (*γ*: ≥ 32 Hz) waves. Each band indicates a different state of mind in response to any stimuli whether it is external or internal. Audio, as well as video, can be stimuli responsible for the activation of state of mind [32]. Although listening to music has enormous effect on the stress reduction, a definite conclusion of its effect cannot be drawn.

To the best of our knowledge, this is the first work that presents the musical intervention impact with varying spectral centroid and lyrics in emotion domain applied for stress relief in male and female categories. Motivation for carrying out this work is to identify an intensity of provoked stress in real-time environment. The contribution of this study is as follows:

- Identified an appropriate band and region where the impact of stimuli is more significant and requires to dig deep to analyze reduction in stress level.
- To analyze the impact of Hindi music with lyrics and varying spectral centroid's impact on stress relief of male and female categories, the EEG signals of the healthy participants (male and female) have been recorded in our university laboratory setting using RMS EEG device. It is hypothesized that musical interventions used would reflect in recorded EEG signals and would help us to analyze the stress impacts in the light of variations in data. In this, four musical interventions have been considered whose goal is to analyze the nonmusical target. Eight features are extracted and fed to the classifier, that is, support vector machine (SVM) for the classification of the music intervention impact on male and female categories. It has been analyzed from experiments that males are more inclined towards triggered music intervention (MI-1, MI-2, and MI-3) as compared to females aged 18–25.

The study is designed in the four sections as follows: Section 2 discusses the related work, whereas Section 3 describes the material and method.  Section 4 gives detailed information about the experiment results of the classification of the impact on the stress level in the male and the female category in response to different types of music along with the discussion of results, whereas Section 5 deals with the conclusion.

## 2. Related Work

According to the authors of reference [33], the left hemisphere's activities are taken over by the right hemisphere pointing to the regions of the stress detection. The beta and alpha bands denote the consciousness level, whereas delta and theta refer to the unconscious state of mind [34]. The prominent beta wave frequencies are known to be the indication of the stress and anxiety levels. Not only various features but also a number of classification methods have been employed by the researchers in the process of assessing the stress from the EEG brain signals. Researchers have used various features in order to assess the stress level using EEG signals such as frequency band power, peak frequency in alpha band, cross correlation between band powers, and Hjorth parameters, which are time-based characteristics of EEG waveform [35]. EEG signal is decomposed to bands with the help of signal processing method of discrete wavelet transformation method [36]. Listening to music initiates a number of cognitive processes in the human brain and is, hence, presumed that stress-related cognitive and psychological responses are influenced by the music [37]. Music listening helps reduce the psychological stress and increase the coping capacity of the stress in an individual [38, 39]. In most of the findings, music listening reduces the stress and anxiety level [40, 41], but not all findings show the reductions in the stress or anxiety level after listening to music [42, 43]. Listening to music has shown an impact on the stress and anxiety, which is an automatic response in any threatening or stressful situation. Music is found to activate the brain activities in terms of intense emotions [44–46]; hence, it has a tendency to change the anxiety levels caused by stressful situations or experiences. Several studies have considered different genres of music impact on the stress level and it has been found that classical music helps more in stress relieving than nonclassical music such as heavy metal and hard rock [40]. Not only music or noise but sedative music and silence also contribute to reducing the stress and help people to relax [47, 48]. Overall, there is merely no difference in the impact of silence, sedative, and simulative music on the stress relief [49]. In reference [50], the authors introduce the various perception of music. Music can be used for acquiring concentration or attention while doing some cognitive activities. In reference [51], the authors find that music has an impact on the performance of every participant while doing some cognitive activities. Not only has this but the results also helped to discriminate the genders of the participants. The difference in the characteristics of music leads to variations in the psychological regulation of the adolescents. The authors use EEG signals to prove this and claim that this conclusion could be used for treating the psychological disorders diseases [52]. In reference [53], the authors studied the EEG signals under the influence of favourite and the relaxed music stimuli and concluded that relaxing music has a better effect than the favourite one even listened for a longer period. EEG is used rapidly to acquire the stress level of an individual by analyzing the brain signals captured. In reference [54], the authors use time and frequency features from the EEG signals and applied supervised learning algorithms in order to get the worker's stress level while working and achieved 80.32% of accuracy. Many researchers have used EEG signals to compute the stress level by analyzing the brainwaves [55–57]. Responsible factors of stress in the job and its impact have been presented [58–60]. Hence, a relevant conclusion cannot be drawn about the effect of music on the brain activity or emotional behaviour of the stress response. Apart from the lack of discovering the impact of music on the stress, the existing literature contradicts the results when compared with the self-reported stress. The limitations that contradict could be a small sample size or different preferred types of music.

## 3. Material and Methods

### 3.1. Material

**Figure 1:**
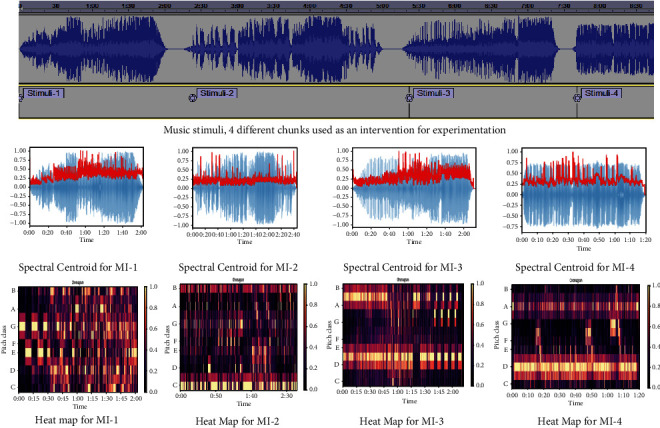
Characteristics of four musical interventions used in study.

Data are acquired as EEG signals in response to the 4 Musical Interventions (MI-1, MI-2, MI-3, and MI-4) with Hindi lyrics and varying spectral centroid feature with a gap of 60 seconds between every intervention, and characteristics of the stimuli used are shown in Figure 1. Features have been extracted from five bands of every channel (14 channels). The feature file is then passed to the classifier and then inferred the results accordingly. A further explanation of the whole process is provided below.

### 3.2. Performance Evaluation Parameters

Statistical parameters sensitivity (Sen), specificity (Spec), and accuracy (Acc) have been computed to measure the performance of classifier. Good performance of a classifier model determines how efficiently classifier determines the correct and incorrect. To measure the performance of the classifier, percentage of correctly classified is defined as a ratio of correctly predicted class to the correctly predicted and incorrectly predicted as shown in the following equations:

(1) $$\begin{array}{c} \text{Sen}=\frac{\text{TP}}{\text{TP}+\text{FN}}*100\text{\%,} \end{array}$$

(2) $$\begin{array}{c} \text{Spec}=\frac{\text{TN}}{\text{TN}+\text{FP}}*10, \end{array}$$

(3) $$\begin{array}{c} \text{Acc}=\frac{\text{TP}+\text{TN}}{\text{TP}+\text{FN}+\text{TN}+\text{FP}}*10, \end{array}$$

where TP is the correct input correct output (CICO) (appropriate), FP is the incorrect input correct output (IICO) (misclassified), TN is the incorrect input incorrect output (IIIO) (appropriate), and FN is the correct input incorrect output (CIIO) (misclassified).

### 3.3. Methods

#### 3.3.1. Data Acquisition

**Figure 2:**
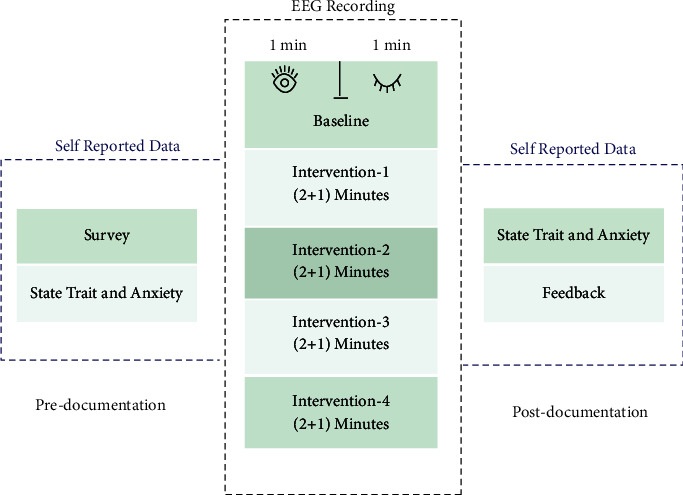
Flow of data acquisition.

A total of 18 healthy participants, 9 male and 9 female, volunteered for the project were aged between 18 and 25 as this age group is most vulnerable in order to cope with stress. The consent form has been signed by all the volunteers and preliminary demonstration has been presented to make them acquainted with the entire procedure. The information such as name, age, gender, and self-reported emotions before listening/after listening to types of songs of the participant was collected through a set questionnaire. Subjects were not trained in music. STA questionnaire has also been filled out by the participant before/after the conduct of study to support the findings. The complete study protocol is as shown in Figure 2. Four types of music interventions (MI-1 to MI-4) have been used for experimentation where lyrics are in the Hindi language and all subjects involved in this study were well acquainted with the language and musical property considered is varying spectral centroid music is well associated with arousal of emotion. MI details are shown in Figure 1.

**Figure 3:**
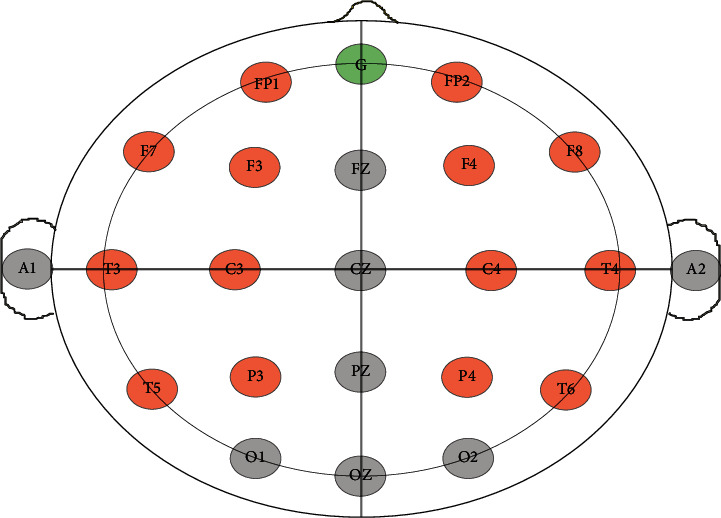
The channel selection.

RMS EEG devices were used to capture the EEG signals of the participants in response to the four MIs with a sampling frequency of 256 Hz. Study involves two trials: trial-0 for the baseline and trial-1 for actual data capture when subjects were exposed to interventions. Duration for trail 0 is 2 minutes (eyes opened/closed 1 min each), and trail 2 is 8 minutes and divided into 4 chunks of 2 min duration in response to 4 MIs as shown in Figure 2. In addition to two trials, the rest time was given to subjects for 1 min in between each MI to avoid any overlapping of data in response to MI but has not been considered for analysis. Postdata recording STA form has been filled out by the volunteers along with the feedback form. The impedance limit set for the data capture is 20 k ohms. The considered channels in the study are C3 and C4 of the central region, F3, F4, F7, F8, FP1, and FP2 of the frontal region, P3 and P4 of the parietal region, and T3, T4, T5, T6 of the temporal region, as shown in Figure 3. Channel selections provide the 360-degree view of arousal/nonarousal of the signals in a particular region in response to interventions presented.

#### 3.3.2. Preprocessing

**Figure 4:**
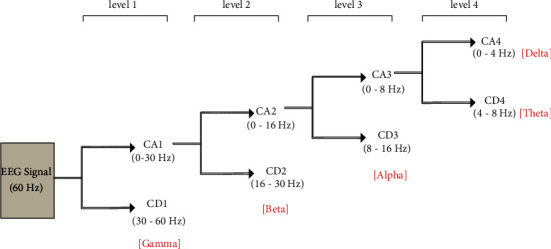
Decomposition of EEG signal into bands.

Frequency till 60 Hz has been filtered by a butterworth filter. The device used in experimentation has not been associated with any filter, and thus it has generated the noise of 50 Hz and has removed the noise from the data before generating the bands. After filtering, the data then passed through the discrete wavelet transformation using wavelet db7 to decompose the data into the bands as gamma waves (30–60 Hz, excluded 50 Hz), beta waves (16–30 Hz), alpha waves (8–16 Hz), theta waves (4–8 Hz), and delta waves (0–4 Hz) for each channel. The decomposition is shown in Figure 4.

#### 3.3.3. Feature Extraction

Features were extracted from five bands of every channel for an entire individual participant in response to four musical interventions. From EEG signals, we can draw an ample amount of information relevant to the functional patterns of the brain. The channels taken for the study are 14, which are C4 and C3 of the central region, F3, F4, F7, F8, FP1, and FP2 of the frontal region, P3 and P4 of the parietal region, and T3, T4, T5, and T6 of the temporal region. Hjorth parameter and statistical features are used to quantify the stress level in male and female categories [61, 62]. The activity parameter represents the signal power and the variance of a time function. This can indicate the surface of the power spectrum in the frequency domain. This is represented by equation (4). The mobility parameter represents the mean frequency or the proportion of standard deviation of the power spectrum as in equation (5). The complexity parameter represents the change in frequency as in equation (6):

(4) $$\begin{array}{c} \text{Activity}=\text{var}\left(y\left(t\right)\right), \end{array}$$

(5) $$\begin{array}{c} \text{Mobility}=\sqrt{\frac{\text{var}\left(\text{d}y\left(t\right)/\text{d}t\right)}{\text{var}\left(y\left(t\right)\right)}}, \end{array}$$

(6) $$\begin{array}{c} \text{Complexity}=\sqrt{\frac{\text{Mobility}\left(\text{d}y\left(t\right)/\text{d}t\right.}{\text{Mobility}\left(y\left(t\right)\right)}};\left(t\right), \end{array}$$

where *y(t)* is denoted as a signal.

*(1) Statistical Features*. It includes the feature standard deviation, variance, skew, kurtosis, and mean. Standard deviation measures how spread out the values are and is the square root of the variance and variance is the squared difference of the mean. Skew computes the symmetry of the dataset, whereas kurtosis checks whether the data are heavy tailed or light tailed to a normal distribution. The total sample size for each channel is 256*∗*duration in sec. Thus, for each stimulus response, the dataset handled is of dimension 256 samples per second *∗*120 sec duration *∗*14 channels = 430080 samples for each subject. A total of eight features were extracted from each band of every channel for the entire subjects in the window size of 30 sec for each song. The obtained feature matrix for one band of a channel of single subject and individual song was 4 × 8. The nonparametric Wilcoxon Signed Rank Sum Test was used with *p* ≥ 0.05 and features have been eliminated on the basis of the results. The null hypothesis for the study is there is no significant impact on the brain region as a response to MIs and alternate hypothesis set is intervention of MIs would derive a significant impact on the brain region and would support relieving from stress.

#### 3.3.4. Classification

For classification, the support vector machine (SVM) has been used to perform a binary classification. The kernel used was polynomial. The feature dataset was segregated into training and testing data in the ratio of 8:2, total samples in feature file are 4*∗*8 for each subject/band, thus 80% for training involves approximately statistically significant 2048 samples for training and 512 samples for training at the stimuli level, and then linear classification is performed. Run time for classifier depends upon prominent features in selecting recording like number of spikes. The classifier used in this study is of binary nature and maximum iteration limit = 100 and numerical tolerance = 0.0010. For classification, subsets considered are the baseline vs. male (in response to MIs), baseline vs. female (in response to MIs), and male vs. female (in response to MIs).

## 4. Result Analysis and Discussion

### 4.1. Analysis of Proposed Work

Analysis of proposed work has been carried out at three subsets: level 1, male vs. female; level 2, baseline vs. male; and level 3, baseline vs. female. Commonly in all subsets out of seven extracted features, hjorth activity that signifies power associated with the signal has been found prominent.

For subset baseline vs. female and the impact of an intervention MI-1, baseline signal has shown tight spread in contrast to the response signal for the region's central, parietal, and temporal, whereas exactly reverse case has been identified for frontal regions, which motivates us analyze the statistical comparative statements at channel and band level as well. The impact of an intervention MI-3 and MI-4 on female in contrast to the baseline is more prominent, comparable, and contrast for central, frontal, and temporal regions of the brain, whereas in parietal, it has not shown any variability in acquired data. In central and frontal regions for MI-3, more variation in the median has been captured in contrast to the temporal region and the same results have been captured for intervention MI-4 as well.

**Figure 5:**
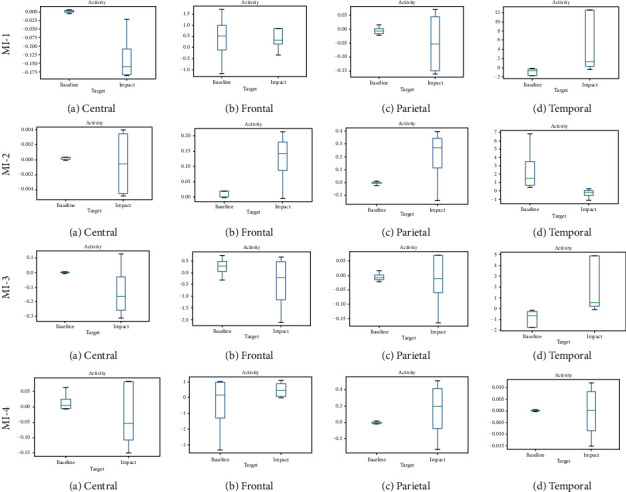
Comparative plots of the impact of interventions (MI-1 to MI-4) on the baseline and male category in (a) central region, (b) frontal, (c) parietal, and (d) temporal region with respect to the hjorth parameter activity.

For subset baseline vs. male and impact of intervention MI-1 in male category, data values are presented in comparative plots as shown in Figure 5 for all four regions (central, frontal, parietal, and temporal). Data variability patterns are different for all regions and the median is also showing a maximum difference in all regions except for the temporal region, reflecting the meaning of a higher impact of MI-1 on the male category. For baseline and impact of MI-2 in the male category, data values for all four regions (central, frontal, parietal, and temporal) are different, and median also exhibits maximum difference in all regions, reflecting the meaning of a higher impact of MI-2 in the male category. The baseline and impact of MI-3 data distributions are different for all region, and the median also exhibits the maximum difference in all regions except for the parietal region, reflecting the meaning of a higher impact of MI-3 in male category.

Similarly for the baseline and impact of MI-4 in the male category, data distributions for all four regions (central, frontal, parietal, and temporal) are different except for frontal region, showing less difference in the distribution pattern of well as the median variation reflecting minimum difference and median variation for all other regions showing maximum difference reflecting the meaning of a higher impact of an intervention MI-4 in male category.

**Figure 6:**
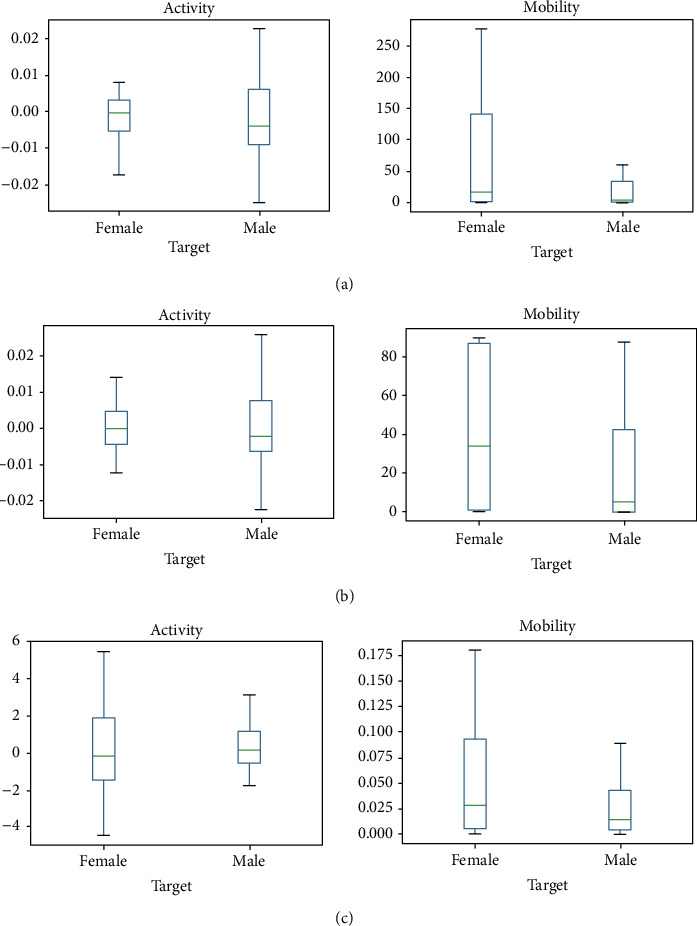
Comparative plots of the impact of MI-2 on male and female category in (a) central region, (b) parietal region, and (c) temporal region with respect to the hjorth parameter activity and mobility.

The representation of the data values of the impact of MI-2 on male and female categories is as shown in comparative plots in Figure 6. The distribution pattern of the box is different for all the regions and the median variation is more, showing the maximum difference in the data values of the central region and parietal region.

Intervention MI-3 has achieved different distribution for the frontal region but is approximately similar to the central region. The median variation is much less, signifying the minimum difference in the data values lies in both central and frontal regions. Impact of MI-4 on subset male vs. female has achieved significant difference for both frontal and central region. The median variation is much less, signifying the minimum difference in the data values lies in both central and frontal regions.

A result obtained and detailed analysis done at channel and band level signify that majorly male is dominant in all scenarios as the result of an impact of interventions. Maximum variability have been found in male categories. Classifier performance according to interventions used for three subsets: (1) male and female, (2) baseline and female, and (3) baseline and male. The objective is to carry the classification for subsets 1, 2, and 3 to identify prominent channels and bands out of the 14 channels and 5 bands for each channel with respect to musical interventions used for experimentation to relate our findings with the state-of-art work in the same domain. In reference [63], the results signify that activation of low-frequency band reflects the reduction in stress level, and results obtained by proposed methodology and interventions are completely in sync by showing activation of low-frequency band delta (0–4 Hz). In reference [64], music has a magical effect on stress reduction and affects highly stress population, and in proposed work, the results have been obtained in the male category rather than female. Interventions used are characteristically different with respect to the spectral centroid and lyrics are related to the emotional domain. For subset 1, in intervention 1, out of 14 channels, only four channels such as C4 (beta), F7 (gamma), FP2 (delta), and P3 (alpha) have achieved acceptable accuracy of above 70% and the rest all channels have been ignored as they have not exhibited prominent results. For subset 1, in intervention 2, out of 14 channels, only 3 channels have achieved acceptable accuracy such as C3 (delta), P3 (gamma), and T5 (theta) and the rest all channels have been ignored as they have not exhibited prominent results. Results of classifier accuracy are listed in Table 1.

Central region, which consists of two electrodes placement, has been analyzed, and the results achieved are for MI-1 at beta band is 72%, for MI-2 at the delta band is 100%, and for MI-3 at delta band is 100%. For MI-4 at theta/gamma band, accuracy obtained is 72%. Maximum accuracy has been achieved from MI-2, and MI-3 spectral centroid is prominent at the beginning and end and MI-3 spectral centroid is prominent at the center, which has a clear indication that in case of MI-2 and MI-3, male and female generate contrast EEG signals, and thus behaviour has been captured by the delta band region.

**Figure 7:**
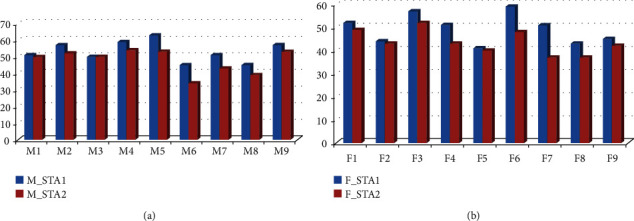
State-trait anxiety analysis for (a) male and (b) female.

In frontal region, for experimentation, 6 electrodes have been placed, three on the left and three on the right side. The observation on the left side of the frontal region has revealed that for MI-1 on the left side of frontal region, maximum accuracy has achieved at FP2 electrode and delta band is 77%, for MI-2, no accuracy has been achieved as compared to other MIs. For MI-3, accuracy achieved is 77% of gamma band and for MI-4, accuracy achieved at gamma band is 72%. The observation on the right side of the frontal region has revealed that except for MI-1, none of the song has reported any difference in the male and female categories. For MI-1 on the right side of the frontal region, maximum accuracy achieved at delta band is 77% and it has been observed at FP2. For parietal section, for MI-1, maximum accuracy achieved at alpha band is 72%, and for MI-2, 77% accuracy has been achieved at gamma band, for MI-3 and MI-4, no significant accuracy has been achieved as compared to MI-1 and MI-2. For temporal region, results have been analyzed for the left and right temporal regions. For left temporal, MI-2 has achieved maximum accuracy of 72% of theta band at T5, whereas other MIs have not shown any significant difference in the generated signal between male and female. For right temporal region, for MI-1, maximum accuracy obtained is 66% of gamma band, whereas accuracy achieved for MI-3 is 66% of beta band and for MI-4 is 61% of the delta band. For analysis of the baseline and MI-1 impact on female, the results retrieved have shown maximum accuracy of 100% at C4 gamma, F3 theta, FP1 beta theta, P3 beta, P4 gamma, T3 gamma, T4 beta, T5 gamma, and T6 gamma. It indicates that signals generated at baseline and MI-1 are not statistically different at central left and frontal right. For analysis of the baseline and MI-2 impact on female, the results retrieved have shown maximum accuracy of 100% C3 beta, F7 beta, FP1 beta, P3 gamma, P4 beta, T3 beta, and T5 gamma. It indicates that signals generated at baseline and MI-2 are not statistically different at central right, frontal right, and temporal right. For the analysis of the baseline and MI-3 impact on female, the results retrieved have shown maximum accuracy of 100% at C4 beta, F7 gamma theta, FP1 beta, FP2 gamma, T3 beta, T4 delta beta, and T5 beta gamma. It indicates that signals generated at baseline and MI-3 are not statistically different at central left and frontal right. Parietal has not shown any impact of music in this region. For the analysis of the baseline and MI-4 impact on female, the results retrieved have shown maximum accuracy of 100% C3 beta, C4 beta, F4 theta, F7 beta, and T5 beta. It indicates that signals generated at baseline and MI-4 are not statistically different at temporal right, and parietal has not shown any impact of intervention in this region. For the analysis of the baseline and MI-1 impact on males, the results retrieved have shown maximum accuracy of 100% at beta C4, beta, gamma F3, theta F4, delta gamma F7, alpha beta FP1, delta gamma FP2, beta gamma P3, theta T4, beta T5, and gamma T6. It indicates that signals generated at baseline and MI-1 are not statistically different at central left, and right parietal region has not shown any significant impact of intervention in this region. For analysis of the baseline and MI-2 impact on males, the results retrieved have shown maximum accuracy of 100% at C3 gamma, F3 gamma, F4 gamma theta, F7 gamma, FP2 beta, P3 beta, P4 beta gamma, T4 delta gamma, and T6 gamma theta. It indicates that signals generated at baseline, MI-2 is not statistically different at central right, and temporal left has not shown any significant impact of music. For analysis of the baseline and MI-3 impact on males, the results retrieved have shown maximum accuracy of 100% C4 beta, F3 alpha beta, FP2 delta, P3 beta, T4 theta gamma, T5 gamma, and T6 gamma. It indicates that signals generated at baseline and MI-3 are not statistically different at central left and parietal right. For the analysis of the baseline and MI-4 impact on males, the results retrieved have shown maximum accuracy of 100% C4 beta, F3 gamma, F4 beta gamma, F7 gamma, F8 beta delta, Fp1 gamma, FP2 beta gamma theta, P3 beta, T4 delta, T5 gamma, and T6 beta. The STA1 score recorded for male is in range of 45–63. The STA2 score recorded is in range of 34–54, which results in standard deviation of 7.12 for male category. The STA1 score recorded for female is in range of 41–59. The STA2 score recorded is in range of 37–52, which results in standard deviation of 6.38 for female category, as shown in Figures 7(a) and 7(b). According to the STA analysis, the deviation in the stress and anxiety level of the male category is found to be more as compared to females. Proposed interventions are most effective for male to reduce stress level as compared to female.

### 4.2. Comparative Study of the Proposed Work with State of the Art

Table 2 has listed the comparative analysis of the proposed work with state of the art. In the proposed work, band-wise and region-wise analysis have been carried out. Central region and delta band have shown major impact to reduce stress level. In references [70–72], interesting linear methods have been proposed.

Challenges in conducting this study are (i) to analyze the particular region that has a higher impact of an intervention, (ii) to deal with variations in intrasubjects of same category as well as different categories, (iii) to deal with intersubject variations because they are related to exact electrode position in multiple sessions, (iv) the impact of this variation has resulted into classification method to generalize for individual subjects as well, and (v) as used intervention has characterized with background music and considered removing and analyzing as suggested in reference [73]. MI details are shown in Figure 1, and survey data include the self-reported data.

## 5. Conclusion

The music interventions with Hindi lyrics and varying spectral centroid in this study have shown the impact on both male and female categories in reducing the stress, but higher impact has been observed in male category. Power associated with signals for delta band of central and frontal region of male category is more dominant in contrast to the female category for first three MIs and differentiated male and female categories. Delta, beta, and gamma bands' activation has been observed dominantly with respect to interventions in the male category, whereas only beta and gamma bands have been observed in the female category. Nonprominent delta band in the female category might be because of the mild impact of musical intervention to invoke the relaxation in the female category, and prominent visibility of delta wave in the temporal region for the male category gives an insight into mindful state in response to music interventions. In future, experimentation will be carried out with a greater number of subjects.

## Figures and Tables

**Table 1:** Classification results for subset1 (male vs. female), subset 2 (baseline vs. female), and subset 3 (baseline vs. male).

Subset	Intervention	Channel	Bands	Accuracy	Sen	Spec

1	MI-1	FP2	Delta	77.78	100	60
	MI-2	C3	Delta, gamma	100, 83.33	100, 66.67	100, 100
	MI-3	C3	Delta	100	100	100
	MI-4	C4, F3	Theta, gamma	72.22	100, 0	100

2	MI-1	C4, P4, T3, T5, T6	Gamma	100	100	100
	MI-2	C3, F7, FP1, P4, T3	Beta	100	100	100
	MI-3	C4, FP1, T3	Beta	100	100	100
	MI-4	C3, C4, F7, T5	Beta	100	100	100

3	MI-1	C4, F3, T5	Beta	100	100	100
	MI-2	C3, F3, F7	Gamma	100	100	100
	MI-3	C4, P3	Beta	100	100	100
	MI-4	C4, P3, T6	Beta	100	100	100
		F3, Fp1, T5	Gamma	100	100	100
		T4	Delta	100	100	100

**Table 2:** Comparative analysis of proposed work with state of art.

Method Year	Number of subjects	Measures	Stimuli	Findings	Algorithm/methodology

2017 [64]	20 college students	EEG signals	Congruent test and incongruent test	The students with higher stress levels have seemed to be affected by the impact of music and the mean beta absolute power ratio increased by 0.246	Mean beta absolute power ratio
2018 [13]	80 infants	Heart rate, oxygen saturation, and neonatal infant pain scale (NIPS)	3 music interventions	The heart rate and pain perception are found to be reduced and oxygen saturation increases in the infants	Statistical analysis (ANOVA test)
2016 [25]	159 patients	State-trait anxiety inventory scale	Background music	Significant reduction found in the anxiety and stress level of the patients	Statistical analysis (Scheffe's test)
2018 [65]	7 subjects	EEG signals	Music	The model prediction for the participant's mental state has accuracy of 80% and with a small MSE loss up to 0.0882	Deep learning
2018 [63]	15 (average age 20.5 years)	EEG signals	15 different music	The findings during listening to music were increased in the spectral power in alpha band and decrease in high-frequency beta and gamma bands	Support vector machine
2019 [66]	15 males and 15 females, aged 20–35 years	EEG signals	4 English music tracks of metal, rock, electronic, and rap genres	Urdu music tracks have more impact on stress reduction than English music tracks	Minimal sequential optimization, stochastic gradient descent, logistic regression, and multilayer perception
			5 Urdu music tracks of famous, melodious, patriotic, qawali, and ghazal genres	Females were more affected by the music than males	
[67–69]	Disabled cancer patients	EEG signals	Music	Effect on frontal lobe	Statistical test
Proposed	9 males and 9 females	EEG signals	4 musical interventions with Hindi lyrics and varying spectral centroid characteristics	Delta band is active for male, which is a clear indication of mindful state as an impact of musical intervention for MI-1, MI-2, and MI-3^∗∗∗∗^	Support vector machine

## Data Availability

Dataset will be provided on request.
